# Applicability of the Instrumented Pendulum Test for Assessing Limb Viscoelastic Properties in Neurological and Internal Diseases: A Narrative Review

**DOI:** 10.3390/life15040535

**Published:** 2025-03-24

**Authors:** Maria Stella Valle, Matteo Cioni, Cristina Russo, Lucia Malaguarnera, Antonino Casabona

**Affiliations:** 1 Laboratory of Neuro-Biomechanics, Section of Physiology, Department of Biomedical and Biotechnological Sciences, School of Medicine, University of Catania, 95123 Catania, Italy; mcioni@unict.it (M.C.);; 2 Section of Pathology, Department of Biomedical and Biotechnological Sciences, School of Medicine, University of Catania, 95123 Catania, Italy; cristina.russo@unict.it (C.R.); lucmal@unict.it (L.M.)

**Keywords:** instrumented pendulum test, stiffness, viscosity, damping, hypotonia, hypertonia, spasticity, chronic obstructive pulmonary disease, rheumatoid arthritis, Down syndrome

## Abstract

Background: The pendulum test was first introduced by Wartenberg as a clinical tool for neurological examination in patients with hypertonia. It was later instrumented to measure the kinematic parameters of gravity-imposed knee movements in patients with spasticity. More recently, the instrumented pendulum test has enabled the quantification of stiffness, viscosity, and damping in both the lower and upper limbs across various neurological and internal diseases. Objective: To highlight the utility of the instrumented pendulum test as a valuable tool for the quantification of stiffness, viscosity, and damping of knee and elbow joints within a clinical setting. Design: Narrative review. Methods: A comprehensive search was conducted using PubMed/MEDLINE, focusing on the terms “pendulum test” combined with “viscosity”, “stiffness”, and “damping”. Results: The instrumented pendulum test effectively quantifies stiffness, viscosity, and damping of the knee and elbow across various conditions, including rheumatic diseases, chronic obstructive pulmonary disease, hypertonia, and hypotonia. Studies have also demonstrated correlations between these non-neural parameters and factors such as age and disease severity. Conclusions: Findings suggest that the instrumented pendulum test could serve as a valuable tool in clinical decision-making for targeted pharmacological treatments, such as botulinum toxin-A or hyaluronidase injections for spasticity, as well as interventions for myofascial system disorders.

## 1. Introduction

Muscle stiffness and viscosity are two key biophysical parameters associated with the serial and parallel elastic components of the musculoskeletal system, primarily contributing to its passive mechanical behavior. Together with the active components of the muscle-tendon complex, these elements form the “three-element model” proposed by Hill [1]. The interplay of these components ensures the stability of the muscle-tendon unit and, consequently, joint functionality. Recent findings by Kodama et al. [2] have highlighted the role of fascia in contributing to stiffness and viscoelasticity.

Stiffness refers to the resistance of structural elements, including muscle, fascia, and peri-articular tissues, to elongation. Research has shown a curvilinear relationship between muscle force and length during passive stretching [3]. Pathological conditions or a sedentary lifestyle that compromise the elastic properties of muscles can result in increased tightness. For example, patients with post-stroke spasticity exhibit altered biophysical characteristics in their lower limb muscles [4], leading to heightened passive resistance to angular displacements at the joint and abnormal torque-angle relationships [5].

Viscosity in fluids, which generates resistance proportional to deformation rate, decreases with rapid flow (shear thinning). Meyer et al. [6] demonstrated that muscle viscosity exhibits similar pseudoplastic behavior. Muscle viscosity is influenced by physical and metabolic factors, including the number of attached cross-bridges, inorganic phosphate concentrations, and mechanical proteins like collagen and titin [7,8,9]. Additionally, hyaluronic acid plays a critical role, as its increase in connective tissue inhibits the sliding of fascial collagen fibers between layers [10]. These factors collectively impact the muscle’s ability to relax and generate maximal tension [6], with metabolic variations playing a significant role in muscle fatigue and reduced cross-bridge detachment rates [11]. Variations in muscle tissue viscosity can profoundly influence physiological function, including energy dissipation and tension regulation. This adaptability helps maintain controlled and smooth movements while preventing excessive stress on the musculoskeletal system.

Both the knee and elbow are complex anatomical structures comprising bones, synovial capsules, ligaments, tendons, and muscles. During joint displacement, each of these components contributes to the resistive forces underlying stiffness and viscosity [12]. Aging and pathological conditions lead to a loss of elasticity, increased fibrous connective tissue, and higher stiffness in muscles and joints [13]. Bohinc et al. [14] investigated the passive stiffness and viscosity of the knee using an instrumented pendulum test on a cadaver. Gradual removal of tissues around the knee resulted in decreased stiffness and viscosity, with muscles and tendons accounting for 40% of the damping effect, ligaments for 20%, and skin for 10%.

Lin et al. [15] used the instrumented pendulum test on the elbow to analyze stiffness in 192 subjects, finding minimal effects of age and gender on these properties. Mechanical characteristics remained stable up to the age of 70, with no significant differences between men and women of comparable body weight.

Given these findings, the quantification of stiffness and viscosity in the upper and lower limbs should be a primary goal in precision rehabilitation medicine, particularly during aging and in pathological conditions linked to neurological or internal diseases. Two primary methodologies are available for the measurement of these parameters in clinical settings: the instrumented pendulum test and shear wave elastography. The instrumented pendulum test quantifies stiffness and viscosity dynamically during passive movements, while shear wave elastography measures these parameters at rest by assessing shear-wave dispersion and attenuation [16]. Both approaches are non-invasive and time-efficient, but the pendulum test uniquely captures dynamic mechanical properties, providing valuable insights into functional biomechanics.

This narrative review aims to consolidate and present evidence on the clinical utility of the instrumented pendulum test in quantifying limb stiffness and viscosity in patients with neurological and internal pathologies. The objective evaluation of stiffness and viscosity in a clinical environment could be useful to address more specific intervention on spastic muscles to be treated with botulinum toxin-A or with hyaluronidase injections according to the “hyaluronidase hypothesis” [17]. In fact, this hypothesis posits that hyaluronic acid accumulation within the extracellular matrix of muscle leads to increased stiffness [18]. Since a tight myofascia might cause muscular stiffness and viscosity [2], a further application could be in the myofascial system disorders to allow an accurate rehabilitative intervention.

## 2. Methods

### 2.1. Search Criteria

A comprehensive search was conducted on PubMed/MEDLINE up to January 2025. The search terms included “pendulum test”, combined with “viscosity”, “damping”, and “stiffness.” Titles, abstracts, and full texts were screened using the following criteria.

### 2.2. Inclusion Criteria

Studies were included if they were written in English, focused on human subjects, provided clinical associations between “pendulum test” combined with “viscosity”, “damping”, and “stiffness”, and reported detailed interventions.

Selected studies were further reviewed to ensure the use of mathematical models for estimating stiffness, viscosity, and damping, with references to foundational works such as Badj and Bowman [19] and Lin and Rymer [20]. No restrictions on publication date were applied.

### 2.3. Exclusion Criteria

Studies such as literature reviews, case reports, letters to the editor, clinical observations, authors’ personal opinions, animal experiments, meeting summaries, and any study that was not peer-reviewed were also excluded. Articles based on mathematical methods without clinical applications were not considered.

### 2.4. Data Extraction and Analysis

Titles and abstracts were independently reviewed by two authors (C.M. and V.M.S.) for relevance. A third reviewer (C.R.) critically evaluated discrepancies, with consensus achieved through discussion. The quality of the studies was assessed according to the Standards for Reporting Qualitative Research (SPQR) [21].

### 2.5. Instrumentation

Precise joint angle measurement is critical in clinical settings, particularly for identifying abnormal patterns and characterizing impairments. The pendulum test employs various tools, from compact electro-goniometers that convert joint angles into electrical signals [22] to advanced technologies like ultrasonic devices [23], optoelectronic systems [24], and inertial sensors (IMU) [25]. These modern technologies, overcoming earlier two-dimensional limitations [26], have become increasingly popular in motion analysis.

### 2.6. Methodology of the Instrumented Pendulum Test

#### 2.6.1. Lower Limb Evaluation

**Figure 1:**
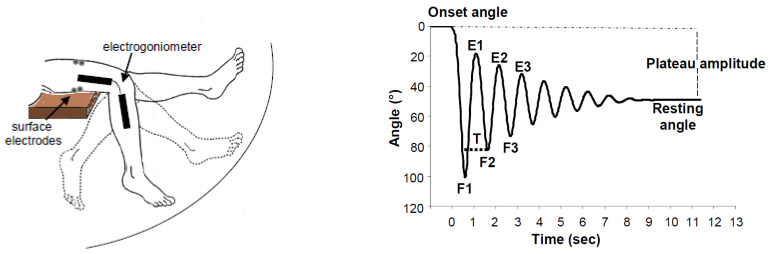
(**Left panel**): passive oscillatory movements during the pendulum test. (**Right panel**): typical normal knee flexion-extension angular response showing onset angle, resting angle, the first three peak flexion angles (F1, F2, F3), the first three extension angles (E1, E2, E3), plateau amplitude, and the period of the first cycle (T).

For the lower limb, subjects are typically positioned on an examination table or a chair and instructed to relax completely. While healthy individuals generally find relaxation easier, some patients may struggle to achieve sufficient relaxation. The test involves the examiner raising the subject’s leg to its maximum horizontal extension. Once relaxation is confirmed, the examiner abruptly releases the leg, allowing it to swing passively under gravity. The leg undergoes several damped oscillations, eventually coming to rest. In normal cases, 5–7 oscillations occur before the leg settles at 75–90 degrees of flexion [27,28]. The first swing is typically the largest, with subsequent oscillations diminishing in amplitude. The first swing excursion is considered a strong predictor of spasticity severity [29,30]. Key kinematic parameters relevant for clinical evaluation include the relaxation index (RI), plateau amplitude (PA), and resting angle (RA) (Figure 1).

#### 2.6.2. Upper Limb Evaluation

For the upper limb, Lin et al. [31] adapted the pendulum test using a setup with a shaft, a predesigned chain, a weight, and wrist fastening. Upon releasing the chain, the forearm swings passively while an electro-goniometer measures elbow angles in the sagittal plane. The kinematic parameters are the same as those used for the knee.

### 2.7. Estimation of Stiffness, Viscosity, and Damping

Kinematic and anthropometric data are used to estimate stiffness (*K*), viscosity (*B*), and damping (*ζ*) for the first three knee joint oscillations [19,20]. The pendulum motion is modeled by the following equation:

$$J\ddot{\theta }+B\dot{\theta }+K\theta +mgl_{c}sin\theta =0$$

where *θ* represents the angle of leg oscillation with respect to the knee; *J* is the moment of inertia of the leg-foot complex with respect to the axis of rotation around the knee; *B* is the viscosity coefficient; *K* is the stiffness coefficient; and the *mgl_c_* factor represents the moment of rotation of the knee produced by gravity (gravitational torque), where *m* is the mass of the leg-foot complex; *l_c_* is the distance between the center of mass (COM) of the leg-foot complex and the axis of rotation around the knee; and *g* is the gravity acceleration. The mass (*m*) is estimated as a percentage of total body weight [32], while *l_c_* is measured as the distance from the fibular head to the ground.

The damping coefficient reflects the speed at which the joint reaches its resting state. In an underdamped oscillation, the system decays slowly, allowing oscillations to occur with gradually decreasing amplitude over time, whereas in an overdamped oscillation, it decays rapidly, with minimal or no oscillations. The damping coefficient reflects the changes in the viscosity component of viscoelastic passive joint resistances: viscosity decreases as the damping coefficient decreases [20]. The damping coefficient (*B*) was estimated by computing the damping ratio (*ζ*) and the natural frequency (*ω*) obtained from the data of each trial [33]:

(1) $$\zeta =\sqrt{\frac{{\left(lnD\right)}^{2}}{{4\pi }^{2}+{\left(lnD\right)}^{2}}}$$

where *D* = θ_1_/θ_2_, that is, the ratio of peak angles between two consecutive cycles.

(2) $$\;\omega =\frac{2\pi }{T}$$

where *T* is the period of one cycle.

Using Equations (1) and (2), the value of the damping coefficient (*B*) is obtained as follows:

(3)  B=2·ζ·ω·J

where *J* is the sagittal moment of inertia applying to the leg-foot complex rotation around the knee axis.

The coefficient of stiffness (*K*) is obtained as follows:

(4) K=J·ω

This approach provides a detailed assessment of the dynamic properties of limb stiffness, viscosity, and damping, offering valuable insights into neuromuscular and biomechanical health.

## 3. Results

**Figure 2:**
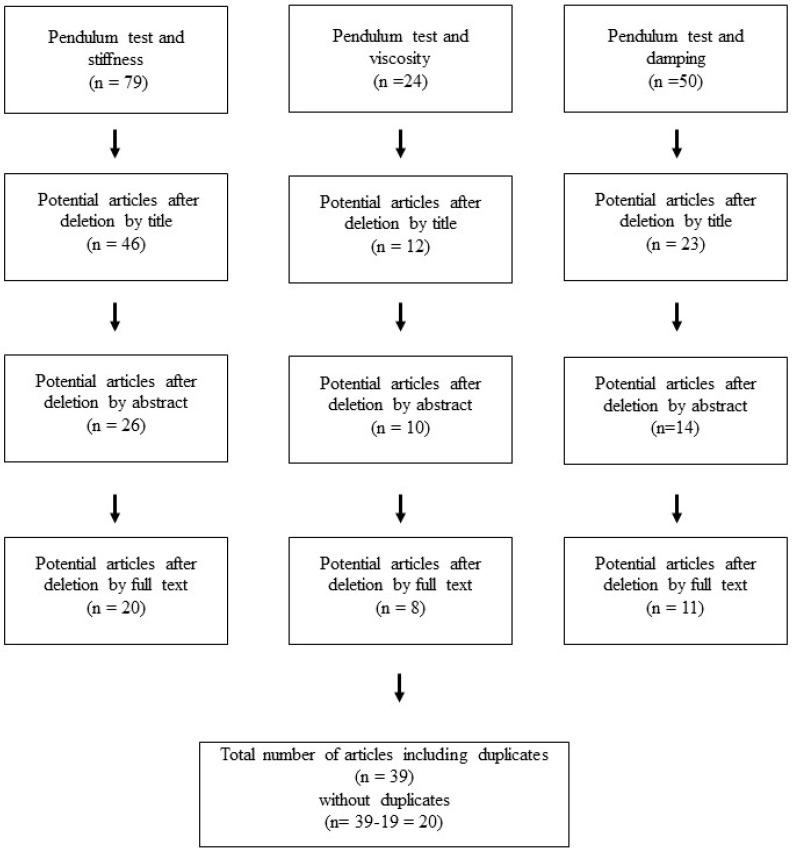
Flow diagram of included articles.

A total of 79 studies were initially identified for “pendulum test and stiffness”, while 24 studies were identified for “pendulum test and viscosity”, and 50 studies were identified for “pendulum test and damping”. Following a review of titles, abstracts, and full texts, 59 studies were excluded for “pendulum test and stiffness”, 16 were excluded for “pendulum test and viscosity”, and 39 were excluded for “pendulum test and damping”. After removing duplicates, 20 articles met the inclusion criteria (Figure 2), and the selected studies are reported in Table 1.

### 3.1. Internal Diseases

#### 3.1.1. Rheumatic Diseases

The instrumented pendulum test has been applied in internal diseases, including rheumatoid arthritis (RA). RA is a chronic inflammatory disease affecting 0.1–2.0% of the global population [45]. Symptoms include pain, swelling, and joint stiffness, with stiffness serving as a key diagnostic criterion [46]. Wright and Johns [47] first attempted to quantify patient-reported stiffness using a finger arthrography based on the principle of the pendulum. Later, Such et al. [48] adapted this method to the knee, demonstrating data reproducibility in healthy subjects. Helliwell et al. [49] highlighted discrepancies between patient-reported stiffness and measurable biophysical parameters, emphasizing the need to quantify stiffness independently.

Valle et al. [23] analyzed knee biomechanics in RA patients using the instrumented pendulum test, correlating increased stiffness (*p* < 0.001) with disease severity (R^2^ = 0.68). Regression analysis suggested stiffness as a predictor of disease severity. In osteoarthritis, Burks and Keegan [34] found reproducible stiffness and viscosity coefficients but no significant correlation with self-reported symptoms, echoing Helliwell’s earlier findings [49]. In fibromyalgia, Wachter et al. [35] reported increased damping of muscle tension during the pendulum test without heightened EMG activity, attributing the changes to non-neural factors.

#### 3.1.2. Chronic Obstructive Pulmonary Disease (COPD)

COPD is a prevalent disease characterized by a chronic inflammatory response affecting the airways, lungs, and skeletal muscles, particularly in the lower limbs [50,51]. Patients with COPD often experience extra-pulmonary functional decline, leading to reduced mobility and balance, which significantly increases the risk of falls. Recent research by Yentes et al. [51] highlighted the value of biomechanical analysis in predicting hospital readmissions, assessing disease severity, and objectively monitoring patients. Earlier, Valle et al. [36], using the instrumented pendulum test, demonstrated changes in the viscoelastic properties of the knee caused by COPD. They found that patients with COPD exhibited reduced knee stiffness and viscosity compared to healthy controls, attributed to dysfunction in connective tissues integrated within the muscle structure, tendons, ligaments, and noncontractile sarcomere proteins. Supporting these findings, Deng et al. [52] utilized ultrasonic elastography on the rectus femoris muscle at rest and confirmed observations of Valle et al. [36], showing that the m. rectus femoris in COPD patients is less stiff than in healthy controls. Furthermore, their study revealed an inverse association between muscle stiffness and lung function, muscle strength, exercise tolerance, and dyspnea severity.

### 3.2. Neurological Diseases

#### 3.2.1. Hypertonia

Wartenberg [27] developed the pendulum test as a clinical tool to broadly quantify muscle tone. He observed that rigidity of extrapyramidal origin primarily influences the quantity of pendulous movements (number and duration), whereas rigidity of pyramidal origin affects the quality of limb oscillations, altering their profile. Additionally, hypotonia was noted to increase the swinging time and number of oscillations, influencing the quantity of movement.

Subsequent advancements incorporated electrogoniometers into the pendulum test, enabling precise quantification of hypertonia through kinematic parameters such as angles, acceleration, and velocity. These advancements facilitated the calculation of indices like the relaxation index and plateau index, which were applied to adults with pyramidal or extrapyramidal hypertonia [53,54] and children with cerebral palsy [29,55]. A significant methodological shift was introduced by Lin and Rymer [20], who aimed to understand the non-linear behavior of the pendulum test. Studying three patients with spastic hemiparesis, they incorporated variables such as intrinsic mechanical properties of the leg, spasticity severity, and limb positioning into a mathematical model. Their work advanced the pendulum test from a second-order linear model, as proposed by Badj and Bowman [19], to a non-linear model of motion. Despite the acknowledged significance of quantifying biophysical muscle parameters, such as stiffness and viscosity, as non-neural determinants of spasticity [4], research in this area has been limited and yielded inconsistent findings. Le Cavorzin et al. [22], comparing patients with spasticity of various origins to a control group, found significantly elevated viscosity coefficients in the spastic group, while stiffness remained unchanged. In contrast, Joghtaei et al. [37], studying patients with paraplegia due to spinal cord injury, observed a significant increase in stiffness coefficients but no alterations in viscosity or damping. Bianchi et al. [38] investigated patients with spastic paraparesis or paraplegia resulting from progressive multiple sclerosis. They observed increased stiffness and viscosity coefficients in three out of six patients compared to controls. Notably, both parameters returned to normal levels following botulinum toxin type A treatment. This study also highlighted the significant variability in these parameters, likely attributable to the inherent variability of the underlying pathology. Huang et al. [39] concluded that the instrumented pendulum test applied to the elbow lacks sufficient sensitivity to differentiate stiffness and viscosity coefficients between patients with post-stroke or Parkinson’s disease. Similar limitations were observed by the same authors in patients with acute cerebellar stroke [40].

#### 3.2.2. Hypotonia

The pathophysiology of hypotonia in Down syndrome remains poorly understood. It may stem from abnormal supra-segmental regulation of muscle tone or dysfunction in striatal muscle activity. Existing literature provides evidence against defective central control of spinal excitability [56] but lacks support for the converse hypothesis.

Genetic studies suggest that an overexpression of collagen molecules may underlie both congenital heart defects and muscle hypotonia in Down syndrome. Collagen, a trimeric extracellular matrix protein, is crucial for the development of skeletal and cardiac muscles [57,58]. Overproduction of collagen is thought to result from gene overdosage at chromosome 21, specifically the COL α1(VI) and α2(VI) chains. Additionally, the COL6A3 gene, which encodes the α3(VI) chain, is located on chromosome 2. Clinically, this overproduction manifests as muscle hypotonia in all infants with Down syndrome and atrioventricular defects in approximately 70% of cases [59].

While muscle hypertonia (a positive sign) has been extensively studied and quantified, muscle hypotonia (a negative sign) remains challenging to measure directly. However, biophysical properties such as stiffness and viscosity can be investigated to improve the understanding of its underlying mechanisms. Ferreira et al. [41] examined the effects of body position on kinematic parameters in adults with Down syndrome, reporting that the damping coefficient was lower in the upright position compared to inclined and supine positions. Earlier, Casabona et al. [33] investigated joint viscoelastic properties in adults with and without Down syndrome, finding reduced stiffness, though not significantly, due to high interindividual variability and a significantly decreased damping coefficient. This reduction resulted in increased amplitude and angular velocity of leg movement in individuals with Down syndrome compared to controls. The authors proposed that a reduced damping coefficient is indicative of muscle hypotonia, which, along with ligament laxity, is characteristic of Down syndrome.

In another pathological condition, type 2 diabetes, patients with polyneuropathy were evaluated using the instrumented pendulum test at elbow [42]. The authors found that the damping coefficient was decreased, while the stiffness coefficient did not differ compared to the control group. These observations suggest that the viscous properties are altered in this pathology and may contribute to the hypotonia observed in the upper limbs.

These findings highlight the importance of further studies on the biophysical properties of hypotonic muscles to advance our understanding of this condition.

### 3.3. Neural Adaptive Mechanisms and Their Influence on Lower Limb Stiffness

The instrumented pendulum test is commonly employed to assess passive joint properties in both healthy and clinical populations. To isolate the influence of kinematic variations, surface electromyography (sEMG) is typically recorded from major muscles surrounding the joint during the test. In healthy individuals, no significant muscle activation is typically observed before or during the pendulum swing. However, certain populations exhibit atypical sEMG activity, such as a peak immediately after limb release or sustained activity throughout the oscillation cycle [33,43,44]. One possible explanation for this phenomenon in individuals with Down syndrome is a compensatory mechanism to counteract joint ligament laxity. This may involve modulating the activity of individual muscles or co-activating synergistic muscles around the joint [33]. For instance, a pronounced phasic sEMG activity during the initial leg flexion, leading to a deceleration of the limb, is often observed, followed by sustained tonic activity [33]. Moreover, the modulation of this muscle response during the initial flexion demonstrates significant age-related variability [43]. Specifically, while the overall amount of rectus femoris sEMG activity (measured as area) was similar in adolescent and adult individuals with Down syndrome, the timing of activation differed. Adolescents exhibited earlier activation peaks (below 50 ms or between 50 and 100 ms), while adults displayed longer muscle response latencies (between 50 and 100 ms or over 100 ms). These variations in muscle response latencies may provide valuable insights into the influence of supraspinal neuronal mechanisms on joint stability, both in the upper [60] and lower limbs [33], suggesting a potential role for transcortical reflexes.

Another example of muscle parameter adaptation is observed in individuals with flaccid paraplegia due to spinal cord injury. Casabona et al. [44] investigated the effects of functional electrical stimulation (FES) training of varying durations (20 min vs. 40 min) on knee joint kinematics, dynamics, and electromyography. Before FES training, these patients exhibited large amplitude oscillations, particularly during the initial flexion (F1). A significant modulatory effect was observed following 20 min of FES training, characterized by reduced angular excursions, increased stiffness, and augmented muscle activity. No significant changes in viscosity were observed across all factors [44].

### 3.4. Methodological Limitations and Proposed Guidelines

As shown in Table 1, most studies focus on the use of the pendulum test at the knee. This test is relatively simple and quick when performed by an experienced examiner. The examiner lifts the leg in the sagittal plane and then releases it suddenly and randomly. However, its validity may be affected by examiner inexperience or inconsistencies in test execution. To mitigate the risk of bias due to examiner inexperience, it is important to recognize that the pendulum test is an instrumented clinical assessment. Before performing the test, the examiner should thoroughly evaluate the patient, collect anamnestic data, and conduct a clinical assessment including goniometry to check for ligament laxity with hypotonia or muscle tightness due to dynamic or static contractures, as these factors can influence the limb’s starting position. Additionally, performing a few pre-test trials allows the examiner to identify erratic or non-compliant participation and exclude such subjects, as recommended by Valle et al. [43]. These authors reported that, out of 45 adults and adolescents with Down syndrome, they excluded 15 adults and 10 adolescents due to their inability to relax and low compliance. From our experience, ensuring proper muscle relaxation is crucial. The examiner should not only assess muscle relaxation by feeling the weight of the limb in his/her hand but also by using real-time surface EMG recordings from antigravity muscles, particularly the m. rectus femoris. This allows the examiner to release the limb only when the muscle exhibits baseline activity without any phasic bursts, ensuring more reliable test execution. In this regard, the number of qualified trials reported in Table 2, for each study, is variable but in most cases is between 5 and 10.

A second important and debated issue concerns the influence of body position on variations in kinematic parameters (Table 2). Both Brown et al. [53] and Azevedo et al. [61] reported that, in healthy subjects, different trunk positions (supine, semi-inclined, sitting upright) did not affect the kinematics of the lower limbs. Conversely, Ferreira et al. [41] found that in a semi-inclined position (45° hip flexion), healthy subjects exhibited a higher relaxation index, whereas in a sitting upright position (90° hip flexion), they showed an increased number of swing cycles and a longer duration of the first knee flexion. The authors suggested that body position influences limb kinematics primarily by altering the tension and length of soft knee tissues, such as tendons and ligaments. In line with this, Azevedo et al. [61] reported that, in patients with spinal cord injury, an upright sitting position (trunk at a 60° angle) yielded results comparable to the control group. However, other trunk positions (supine and semi-supine) caused discomfort, pain, or increased blood pressure. As summarized in Table 2, the most commonly used body position appears to be between 40° and 45° relative to the hip. Therefore, when conducting evaluations before and after a pharmacological or rehabilitation intervention, particular attention should be paid to ensuring a consistent trunk position for each subject and across all participants, verified using a goniometer. A final methodological consideration arises from the study by Willaert et al. [62], which suggests a potential improvement in the pendulum test for children with spastic cerebral palsy. Their findings indicate that when the examiner passively moves the leg up and down before releasing it, the range of motion of the first flexion is greater compared to a condition without pre-movement. The authors attributed this effect to the influence of movement history on short-range stiffness, likely due to the thixotropic behavior of spastic muscles.

Since obtaining reliable dynamic data (including viscosity and stiffness) depends on accurate kinematic measurements, Table 3 presents key methodological guidelines derived from the existing literature on this topic.

## 4. Conclusions

In this review, we have highlighted the effectiveness of the instrumented pendulum test as a reliable tool to quantify the in vivo viscoelastic properties of the upper and lower limbs in both healthy individuals and those affected by neurological or internal diseases. Overall, we observed that in all patient groups reported in these studies, it was possible to quantify both stiffness and viscosity. These parameters were increased in patients with spasticity [20,22,31,37,38], while they were decreased in patients with inflammatory connective tissue disorders [23,34,42] or hypotonia [33,43]. Identifying and quantifying these properties, alongside understanding adaptive responses to muscular hypotonia, are critical steps in implementing preventive and rehabilitative strategies.

From a clinical perspective, studies focusing on more homogeneous patient groups, such as those with genetic disorders (e.g., Down syndrome) or rheumatic diseases, have produced more consistent and robust results. Conversely, research involving populations with central nervous system damage has shown divergent outcomes. A plausible explanation is the heterogeneity of these patient samples in terms of lesion characteristics and the time elapsed since the onset of pathology. Thus, future clinical studies should prioritize recruiting neurological patients with more homogeneous disease profiles and duration since onset, while also considering the specifics of their physical therapy regimens. Understanding the viscoelastic properties of muscles has profound implications for elucidating the development of various pathological conditions. Future research utilizing the instrumented pendulum test could expand to explore diseases such as muscular dystrophy, inflammatory myopathies, and metabolic myopathies. These investigations may not only deepen our understanding of these conditions but also pave the way for targeting these biophysical parameters with pharmacological or rehabilitative interventions. By doing so, the viscoelastic properties of muscle could become central to the development of innovative therapeutic strategies aimed at improving quality of life for affected individuals.

## Figures and Tables

**Table 1:** Selected studies from research on MEDLINE.

Study	Design	Objective	Population	Methods	Results
Knee stiffness and viscosity: New implementation and perspectives in prosthesis development [14]	Experimental study	To identify the contribution of periarticular and intraarticular soft tissues to stiffness and viscosity of knee.	One female cadaver	The instrumented pendulum test at knee was executed by a machine vision system equipped with 6 passive infrareds marked. Progressive removal of soft tissues around the knee with contemporary and multiple trial of evaluation.	Knee damping was reduced. The contribution to the knee damping was of 10% for skin, 20% for ligaments and 40% for muscles and tendons.
Gender and age effects on elbow joint stiffness in healthy [15]	Experimental study	To study the relationships among biomechanical parameters and demographic factors at elbow.	192 healthy men (n.111) and women (n. 81) aged 20~70 y.	The instrumented pendulum test at elbow was done by an electrogoniometer to measure number of swings, relaxation index, stiffness coefficient and damping coefficient.	Stiffness and damping coefficients of the elbow joint were similar in men and women when the data were normalized for body weight.
A quantitative analysis of pendular motion of the lower leg in spastic human subjects [20]	Experimental study	To investigate gravity induced oscillations of the lower leg in subjects with and without spasticity.	3 patients with spastic hemiparesis (1 woman and two men aged respectively 36, 79 and 45 y). 1 healthy woman (25 y)	The instrumented pendulum test at knee was done by wearing a plastic splint with a metal rod connected to a potentiometer to record pendular oscillation. Surface EMG recordings from muscles extensors and flexors of knee.	Motion of a spastic limb during the pendulum test follows a nonlinear model with a variance usually exceeding 90%.
Evaluation of pendulum testing of spasticity [22]	Experimental study	To identify which parameters of the pendulum test are sensitive to spasticity.	15 subjects with spasticity (mean age 61.5 ± 9.0 y, 4 females) and 10 control subjects (mean age 32 ± 9.4 y, 3 females), matched for age, sex and morphometric criteria.	The instrumented pendulum test at knee was performed by an electrogoniometer. Surface EMG recordings of m. rectus femoris and m. semimebranosus during pendulum test. Mathematic model of lower limb swinging.	Viscosity coefficient was found to be significantly different (*p* = 0.014) in the group of patients with spasticity in respect to the control group. Elasticity was not significantly different between the two groups (*p* = 0.064).
The pendulum test as a tool to evaluate the passive knee stiffness and viscosity of patients with rheumatoid arthritis [23]	Experimental study	To study the biomechanical changes of knee caused by rheumatoid arthritis	8 women with rheumatoid arthritis (mean age 52 ± 10 y) and 8 healthy women (mean age 49 ± 10.5 y).	The instrumented pendulum test at knee was done with a motion analysis system (Zebris CMS H) to evaluate kinematic parameters and to calculate the viscoelastic properties of knee joint.	Knee stiffness increased significantly in the group of patients in respect to the control group and it was the main factor causing a reduction of swing oscillations. Knee stiffness was significantly correlated to the severity of disease.
Knee joint kinematics of the pendulum test in children with and without Down syndrome [24]	Experimental analytical study	To identify the difference of knee kinematics between children with and without Down syndrome	15 children with Down syndrome (DS) (4 males, mean age 8.46 ± 1.75) and 15 children without DS (7 males, 8.33 ± 1.87)	The instrumented pendulum test at knee was performed by an eight cameras motion capture system (VICON). Measurements of kinematic parameters of pendulum test. EMG recording from m. m. rectus femoris, vastus lateralis and vastus medialis. Calculation of stiffness and damping coefficients.	Authors suggested a greater stiffness of knee joint, in the group of children with DS. Inclusion of an ankle load improved the joint knee stiffness by significantly increasing the number of cycles and the stiffness coefficient in both groups of children with and without DS.
Assessment of Passive Upper Limb Stiffness and Its Function in Post-Stroke Individuals Wearing an Inertial Sensor during the Pendulum Test [25]	Observational analytical study	To identify the correlations between pendulum test parameters and some clinical scales.	7 subjects chronic post stroke (2 men, mean age 51.9 ± 11.2).	The instrumented pendulum test at elbow was executed by a single wearable sensor attached to the forearm. Measurements of kinematic parameters and natural frequency. MAS, MAL and FM clinical scales. Calculation of damping and stiffness coefficients and damping ratio.	Stiffness coefficient was correlated to the natural frequency of pendulum oscillations (r = 0.96, *p* = 0.003). No correlations of stiffness or damping coefficients with clinical scales.
The pendulum test for evaluating spasticity of the elbow joint [31]	Experimental study	To test a modified pendulum test to quantify spasticity at elbow.	11 men with chronic post -stroke condition (mean age 57.7 ± 16.1 y) and 11 able body men (mean age 59.5 ± 11.8 y).	A clock pendulum test with an electrogoniometer was set up to allow a more comfortable posture and an increased inertia. The number of swings, relaxation index, stiffness and damping coefficients were measured.	Damping coefficient and damping ratio increased in the affected side and worsened with the severity of spasticity.
Functional assessments of the knee joint biomechanics by using pendulum test in adults with Down syndrome [33]	Experimental study	To study the biophysical characteristics of hypotonic muscles	10 adults (4 women, mean age 26 ± 5 y) with Down syndrome (DS) and 10 adults without DS (5 women, mean age 24 ± 5 y).	The instrumented pendulum test at knee was done by using an electrogoniometer to assess kinematic and viscoelastic properties of lower limbs in adults with DS.	Damping coefficient was significantly reduced in persons with DS than control group. Stiffness was similar between subjects with or without DS.
Objective measurements of stiffness in knee osteoarthritis [34]	Observational study	To test reliability of pendulum test for evaluation of knee stiffness in older adults with or without knee osteo-arthritis.	41 participants (8 men) (29 with a diagnosis of knee osteoarthritis and 12 without), mean age 67.6 ± 7.6.	The instrumented pendulum test at knee was done by a three cameras motion capture system (VICON). Stiffness and damping coefficients were calculated within and between each group.	Within-participant variability for stiffness and damping was low (respectively 0.55% and 8.92%) whereas between-participant variability was high (respectively 99.45% and 91.08%).
Muscle damping measured with a modified pendulum test in patients with fibromyalgia, lumbago, and cervical syndrome [35]	Observational study	To quantify the muscle tension and in particular muscle damping.	33 able bodied subjects (aged 20–62 yrs), 15 patients with fibromyalgia (aged 41~82 y), 22 with lumbago (aged 27~68 y), 21 with cervicalgia (aged 27~68 y).	The instrumented pendulum test at knee was executed by means of an electrogoniometer. Muscle damping was calculated for each group.	In respect to the control group, in patients with fibromyalgia and in most of patients with chronic lumbago and cervical syndrome, damping values are elevated (overdamping)
Impact of chronic obstructive pulmonary disease on passive viscoelastic components of the musculoarticular system [36]	Experimental study	To quantify the viscoelastic properties of lower limbs of patients with chronic obstructive pulmonary disease (COPD).	11 patients with COPD (5 men, mean age 63.8 ± 11.4)	The instrumented pendulum test at knee was done by an electrogoniometer to evaluate kinematic and viscoelastic parameters of lower limbs and electromyographic reflex responses of m. rectus femoris and biceps femoris caput longus.	Significant reduction of knee stiffness and viscosity coefficients, in comparison with the control group.
Assessment of passive knee stiffness and viscosity in individuals with spinal cord injury using pendulum test [37]	Observational study	Measurements of stiffness and viscosity in patients with spinal cord injury.	15 subjects with paraplegia due to incomplete spinal cord injury (grade C, AI-C) (men 8, mean age 34.60 ± 9.18) and 15 able-body subjects (men 8, 30.66 ± 11.13 yrs).	The instrumented pendulum test at knee was done by using an electrogoniometer to measure viscoelastic parameters of the knee.	Stiffness of subjects with paraplegia was significantly increased in respect to the control group (*p* = 0.01). Viscosity was not significantly different.
Quantitative analysis of the pendulum test: application to multiple sclerosis patients treated with botulinum toxin [38]	Experimental study	To set up a quantitative analytical method to quantify the effects of botulinum toxin type A (BoNT-A) on patients with spasticity.	6 patients affected by progressive multiple sclerosis (aged 29–55 y, 1 male).	The instrumented pendulum test at knee was done with a 3-D motion analysis system (ELITE system). Recordings were done before and after the treatment with botulinum toxin type A.	Before treatment with BoNT-A of knee flexor muscles, both viscosity and stiffness coefficients were significantly higher than the control group. After BoNT-A both knee stiffness and viscosity coefficients were decreased.
Flexor and extensor muscle tone evaluated using the quantitative pendulum test in stroke and parkinsonian patients [39]	Experimental study	To investigate the difference in hypertonia between spasticity and rigidity.	21 subjects with Parkinson’s disease (men 9, mean age 68.3 ± 9.9) and 14 subjects with post-stroke (men 9, mean age 65.1 ± 8.8). Control group was composed by 22 subjects (men 11, mean age 63.2 ± 7.5).	The instrumented pendulum test at elbow was performed by using an electrogoniometer to quantify kinematic parameters, stiffness, viscosity and damping.	Stiffness coefficient was similar between patients with pyramidal or extrapyramidal hypertonia. The instrumented pendulum test does not allow to differentiate hypertonia of different origin. The clinical characteristics of these patients are not reported.
Muscle tone of upper limbs evaluated by quantitative pendulum test in patients with acute cerebellar stroke [40]	Observational study	To quantify the muscle tone of upper limbs.	8 subjects (4 females, mean age 70 ± 8.2 y ) with acute cerebellar stroke.	The instrumented pendulum test at elbow was done by an electrogoniometer to measure number of swings, relaxation index, stiffness coefficient and damping coefficient.	Damping coefficient of the affected side was significantly smaller than that on the intact arm.
Effect of body position and external ankle load on the pendulum test in adults [41]	Experimental analytical study	To test if the body position affects the lower limb kinematics and the estimation of viscosity and stiffness. To investigate the effects of external ankle loads on kinematics of pendulum test.	20 young healthy adults (10 men, mean age 22.4 ± 3.1).	The instrumented pendulum test at knee was done by using 8 cameras motion capture system (VICON). EMG recording from m. rectus femoris, vastus lateralis and vastus medialis. Calculation of stiffness and damping coefficients	Damping coefficient was lower in the upright position than both the supine and inclined positions (*p* < 0.001) Heavier load conditions (3% and 6% of body weight) produced lower stiffness and damping coefficients that 0% load coefficient.
Muscle tone in diabetic polyneuropathy evaluated by the quantitative pendulum test [42]	Case control study	To quantify the muscle tone of upper limbs	n. 53 subjects (mean age 58.4 ± 9.5 y) with diabetic neuropathy and 128 healthy subjects (mean age 58.1 ± 11.1 y).	The instrumented pendulum test at elbow was done by using an electrogoniometer to measure number of swings, relaxation index, stiffness constant and damping coefficient.	Damping coefficient decreased significantly in patients with diabetic neuropathy in respect to the control group. Stiffness constant was not significantly different.
Timing of Muscle Response to a Sudden Leg Perturbation: Comparison between Adolescents and Adults with Down Syndrome [43]	Experimental study	To identify the adaptive response to a sudden lower limb load perturbation in adolescents and adults with Down syndrome.	10 adult subjects with Down syndrome (DS) (age 25.5 ± 3.7 yrs) and 10 adolescents with DS (age 13.9 ± 3.1 yrs).	The instrumented pendulum test at knee was done with an electrogoniometer to evaluate kinematic and viscoelastic parameters and electromyographic reflex responses of m. rectus femoris and biceps femoris.	Significant differences of damping and stiffness were observed between adolescents and adults with Down Syndrome. Different latencies of surface EMG responses of rectus femoris in the two groups.
Effects of Functional Electrical Stimulation Cycling of Different Duration on Viscoelastic and Electromyographic Properties of the Knee in Patients with Spinal Cord Injury [44]	Experimental study	To identify the best temporal dose of Functional Electrical Stimulation (FES).	7 young adults (1 woman, mean age 32.3 ± 4.8) with flaccid paraplegia due to a lesion of spinal cord. AIS-A (5 subjects) and B (2 subjects).	The instrumented pendulum test at knee was done by an electrogoniomter to identify changes induced by functional electric stimulation (FES) on knee angle excursion, stiffness and viscosity.	Stiffness of knee increased statistically both after 20- and 40-min exercises of FES cycling. The best significant functional changes of knee mobility were obtained with the FES dose of 20 min. Viscosity was not significantly modified.

**Table 2:** Methodological parameters of pendulum test at the knee.

Study	Trunk/Hip	sEMG	Trials	Stiffness	Viscosity
	Angle	Muscles	n°	K	B
Knee stiffness and viscosity: New implementation and perspectives in prosthesis development [14]	0°	-	1	Kg m^2^/s^2^	Kg m^2^/s
Evaluation of pendulum testing of spasticity [22]	90°	-	5–10	N/A	N/A
The pendulum test as a tool to evaluate the passive knee stiffness and viscosity of patients with rheumatoid arthritis [23]	40°	-	10	N/rad m^4^	N-m-s/rad
Knee joint kinematics of the pendulum test in children with and without Down syndrome [24]	90°	-	5	N-m/rad	N-m-s/rad
Functional assessments of the knee joint biomechanics by using the pendulum test in adults with Down syndrome [33]	40°	RF	10	N-m/rad	N-m-s/rad
Objective measurements of stiffness in knee osteoarthritis [34]	90°	-	3	N-m/rad	N-m/rad s
Muscle damping measured with a modified pendulum test in patients with fibromyalgia, lumbago, and cervical syndrome [35]	40°	VL, BF	2–3	N/A	N/A
Impact of chronic obstructive pulmonary disease on passive viscoelastic components of the musculoarticular system [36]	40°	RF, BF	10	N-m/rad-Normalized for G.T.	N-m-s/rad
Assessment of passive knee stiffness and viscosity in individuals with spinal cord injury using the pendulum test [37]	45°	-	3	N-m/rad m^4^	N-m-s/rad m^4^
Quantitative analysis of the pendulum test: application to multiple sclerosis patients treated with botulinum toxin [38]	45°	-	56–61	N-m/rad	N-m-s/rad
Effect of body position and external ankle load on the pendulum test in adults [41]	0°, 45°, 90°	RF, VL, VM	5	N-m/rad	N-m-s/rad
Timing of muscle response to a sudden leg perturbation: comparison between adolescents and adults with Down syndrome [43]	40°	RF, BF	10	N-m/rad	N-m-s/rad
Effects of functional electrical stimulation cycling of different duration on viscoelastic and electromyographic properties of the knee in patients with spinal cord injury [44]	45°	RF, BF	10	N-m/rad Normalized for G.T.	N-m-s/rad Normalized for G.T.

RF = Rectus Femoris; VL = Vastus Lateralis; VM = Vastus Medialis; BF = Biceps Femoris; G.T. = gravitational torque; N-m = Newton meters; rad = radiant; N/A = not available.

**Table 3:** Guidelines for the execution of the instrumented knee pendulum test.

	Actions
1	History and clinical evaluation of patients.
2	Body position (trunk-hip angle: between 40° and 45°) with backrest. Arms in lap.
3	The examiner should be in front of the patient.
4	Check for some pain or limitation of knee flexion/extension movements.
5	Explain and allow the patient to practice for some trials.
6	Check for the patient’s compliance and ability to relax.
7	Pre-trial movements before each trial (for patients with spasticity).
8	Online recordings of kinematics by an electrogoniometer, IMU or 2D/3D motion analysis systems.
9	Online recordings of sEMG activity of m. rectus femoris and m. biceps femoris.
10	Collect between 5 and 10 qualified trials.
11	Data elaboration.
12	Report with clinical data, kinematics, sEMG, stiffness, and viscosity.

## Data Availability

References for this review were identified through searches of PubMed for articles published until 2025.

## References

[1] Hill A.V. (1938). The Heat of Shortening and the Dynamic Constants of Muscle. Proc. R. Soc. Lond. B Biol. Sci..

[2] Kodama Y., Masuda S., Ohmori T., Kanamaru A., Tanaka M., Sakaguchi T., Nakagawa M. (2023). Response to mechanical properties and physiological challenges of fascia: Diagnostic and rehabilitative therapeutic intervention for myofascial system disorders. Bioengineering.

[3] Gareis H., Solomonow M., Baratta R., Best R., D’Ambrosia R. (1992). The isometric length-force models of nine different skeletal muscles. J. Biomech..

[4] Dietz V., Quintern J., Berger W. (1981). Electrophysiological studies of gait in spasticity and rigidity. Evidence that altered mechanical properties of muscle contribute to hypertonia. Brain.

[5] Hufschmidt A., Mauritz K.H. (1985). Chronic transformation of muscle in spasticity: A peripheral contribution to increased tone. J. Neurol. Neurosurg. Psychiatry.

[6] Meyer G.A., McCulloch A.D., Lieber R.L. (2011). A nonlinear model of passive muscle viscosity. J. Biomech. Eng..

[7] Mauro A., Adams W.R. (1961). The structure of the sarcolemma of the frog skeletal muscle fiber. J. Biophys. Biochem. Cytol..

[8] Moss R.L., Halpern W. (1977). Elastic and viscous properties of resting frog skeletal muscle. Biophys. J..

[9] Stackhouse S.K., Reisman D.S., Binder-Macleod S.A. (2001). Challenging the role of pH in skeletal muscle fatigue. Phys. Ther..

[10] Amir A., Kim S., Stecco A., Jankowski M.P., Raghavan P. (2022). Hyaluronan homeostasis and its role in pain and muscle stiffness. PM & R.

[11] Zhang L.Q., Rymer W.Z. (2001). Reflex and intrinsic changes induced by fatigue of human elbow extensor muscles. J. Neurophysiol..

[12] Helliwell P.S., Wright V., Radin E.L. (1993). Joint stiffness. Mechanics of Joint: Physiology, Pathophysiology and Treatment.

[13] Timiras P.S., Navazio F.M., Timiras P.S. (2007). The skeleton, joints, and skeletal and cardiac muscles. The Physiological Basis for Aging and Geriatrics.

[14] Bohinc K., Vantur N., Torkar D., Lampe T., Hribernik M., Jakovljević M. (2017). Knee stiffness and viscosity: New implementation and perspectives in prosthesis development. Bosn. J. Basic Med. Sci..

[15] Lin C.C., Ju M.S., Huang H.W. (2005). Gender and age effects on elbow joint stiffness in healthy subjects. Arch. Phys. Med. Rehabil..

[16] Creze M., Soubeyrand M., Yue J.L., Gagey O., Maître X., Bellin M.F. (2018). Magnetic resonance elastography of the lumbar back muscles: A preliminary study. Clin. Anat..

[17] Cowman M.K., Schmidt T.A., Raghavan P., Stecco A. (2015). Viscoelastic Properties of Hyaluronan in Physiological Conditions. F1000Research.

[18] Raghavan P. (2018). Emerging Therapies for Spastic Movement Disorders. Phys. Med. Rehabil. Clin. N. Am..

[19] Badj T., Bowman B. (1982). Testing and modeling of spasticity. J. Biomed. Eng..

[20] Lin D.C., Rymer W.Z. (1991). A quantitative analysis of pendular motion of the lower leg in spastic human subjects. IEEE Trans. Biomed. Eng..

[21] O’Brien B.C., Harris I.B., Beckman T.J., Reed D.A., Cook D.A. (2014). Standards for reporting qualitative research: A synthesis of recommendations. Acad. Med..

[22] Le Cavorzin P., Hernot X., Bartier O., Carrault G., Chagneau F., Gallien P., Allain H., Rochcongar P. (2002). Evaluation de la mesure de la spasticité par le pendulum test [Evaluation of pendulum testing of spasticity]. Ann. Readapt. Med. Phys..

[23] Valle M.S., Casabona A., Sgarlata R., Garozzo R., Vinci M., Cioni M. (2006). The pendulum test as a tool to evaluate the passive knee stiffness and viscosity of patients with rheumatoid arthritis. BMC Musculoskelet. Disord..

[24] Ferreira D.M., Liang H., Wu J. (2020). Knee joint kinematics of the pendulum test in children with and without Down syndrome. Gait Posture.

[25] de Lima M.S.N., Dos Santos Couto Paz C.C., Ribeiro T.G., Fachin-Martins E. (2023). Assessment of Passive Upper Limb Stiffness and Its Function in Post-Stroke Individuals Wearing an Inertial Sensor during the Pendulum Test. Sensors.

[26] Rahimi F., Eyvazpour R., Salahshour N., Azghani M.R. (2020). Objective assessment of spasticity by pendulum test: A systematic review on methods of implementation and outcome measures. Biomed. Eng. Online.

[27] Wartenberg R. (1951). Pendulousness of the legs as a diagnostic test. Neurology.

[28] Oatis C.A. (1993). The use of a mechanical model to describe the stiffness and damping characteristics of the knee joint in healthy adults. Phys. Ther..

[29] Fowler E.G., Nwigwe A.I., Ho T.W. (2000). Sensitivity of the pendulum test for assessing spasticity in persons with cerebral palsy. Dev. Med. Child Neurol..

[30] Szopa A., Domagalska-Szopa M., Kidoñ Z., Syczewska M. (2014). Quadriceps femoris spasticity in children with cerebral palsy: Measurement with the pendulum test and relationship with gait abnormalities. J. Neuroeng. Rehabil..

[31] Lin C.C., Ju M.S., Lin C.W. (2003). The pendulum test for evaluating spasticity of the elbow joint. Arch. Phys. Med. Rehabil..

[32] Winter D.A. (2009). Anthropometry. Biomechanics and Motor Control of Human Movement.

[33] Casabona A., Valle M.S., Pisasale M., Pantò M.R., Cioni M. (2012). Functional assessments of the knee joint biomechanics by using pendulum test in adults with Down syndrome. J. Appl. Physiol..

[34] Burks K., Keegan K. (2006). Objective measurement of stiffness in knee osteoarthritis. Orthop. Nurs..

[35] Wachter K.C., Kaeser H.E., Gühring H., Ettlin T.M., Mennet P., Müller W. (1996). Muscle damping measured with a modified pendulum test in patients with fibromyalgia, lumbago, and cervical syndrome. Spine.

[36] Valle M.S., Casabona A., Di Fazio E., Crimi C., Russo C., Malaguarnera L., Crimi N., Cioni M. (2021). Impact of chronic obstructive pulmonary disease on passive viscoelastic components of the musculoarticular system. Sci. Rep..

[37] Joghtaei M., Arab A.M., Hashemi-Nasl H., Joghataei M.T., Tokhi M.O. (2015). Assessment of passive knee stiffness and viscosity in individuals with spinal cord injury using pendulum test. J. Spinal Cord Med..

[38] Bianchi L., Monaldi F., Paolucci S., Iani C., Lacquaniti F. (1999). Quantitative analysis of the pendulum test: Application to multiple sclerosis patients treated with botulinum toxin. Funct. Neurol..

[39] Huang H.W., Ju M.S., Lin C.C. (2016). Flexor and extensor muscle tone evaluated using the quantitative pendulum test in stroke and parkinsonian patients. J. Clin. Neurosci..

[40] Huang H.W., Ju M.S., Wang W.C., Lin C.C. (2009). Muscle tone of upper limbs evaluated by quantitative pendulum test in patients with acute cerebellar stroke. Acta Neurol. Taiwan.

[41] Ferreira D.M., Liang H., Wu J. (2023). Effect of body position and external ankle load on the pendulum test in adults. Knee.

[42] Lin C.C., Ju M.S., Huang H.W. (2007). Muscle tone in diabetic polyneuropathy evaluated by the quantitative pendulum test. Arch. Phys. Med. Rehabil..

[43] Valle M.S., Cioni M., Pisasale M., Pantò M.R., Casabona A. (2013). Timing of Muscle Response to a Sudden Leg Perturbation: Comparison between Adolescents and Adults with Down Syndrome. PLoS ONE.

[44] Casabona A., Valle M.S., Dominante C., Laudani L., Onesta M.P., Cioni M. (2020). Effects of Functional Electrical Stimulation Cycling of Different Duration on Viscoelastic and Electromyographic Properties of the Knee in Patients with Spinal Cord Injury. Brain Sci..

[45] Almutairi K.B., Nossent J.C., Preen D.B., Keen H.I., Inderjeeth C.A. (2021). The Prevalence of Rheumatoid Arthritis: A Systematic Review of Population-based Studies. J. Rheumatol..

[46] Ropes M.W., Bennett G.A., Cobb S., Jacox R., Jessar R.A. (1959). Revision of diagnostic criteria for rheumatoid arthritis. Arthritis Rheum..

[47] Wright V., Johns R.J. (1960). Physical factors concerned with the stiffness of normal or diseased joints. Bull. Johns Hopkins Hosp..

[48] Such C.H., Unsworth A., Wright V., Dowson D. (1975). Quantitative study of stiffness in the knee joint. Ann. Rheum. Dis..

[49] Helliwell P.S., Howe A., Wright V. (1987). The measurement of stiffness in the rheumatoid hand. Eng. Med..

[50] Jaitovich A., Barreiro E. (2018). Skeletal Muscle Dysfunction in Chronic Obstructive Pulmonary Disease. What We Know and Can Do for Our Patients. Am. J. Respir. Crit. Care Med..

[51] Yentes J.M., Wai-Yan Liu W.Y., Zhang K., Markvicka E., Rennard S.D.I. (2022). Updated Perspectives on the Role of Biomechanics in COPD: Considerations for the Clinicians. Int. J. Chronic Obstr. Pulm. Dis..

[52] Deng M., Zhou X., Li Y., Yin Y., Liang C., Zhang Q., Lu J., Wang M., Wang Y., Sun Y. (2022). Ultrasonic Elastography of the Rectus Femoris, a Potential Tool to Predict Sarcopenia in Patients With Chronic Obstructive Pulmonary Disease. Front. Physiol..

[53] Brown R.A., Lawson D.A., Leslie G.C., MacArthur A., MacLennan W.J., McMurdo M.E., Mutch W.J., Part N.J. (1988). Does the Wartenberg pendulum test differentiate quantitatively between spasticity and rigidity? A study in elderly stroke and Parkinsonian patients. J. Neurol. Neurosurg. Psychiatry.

[54] Jamshidi M., Smith A.W. (1996). Clinical measurement of spasticity usiong the pendulum test: Comparison of electrogoniometric and videotape analyses. Arch. Phys. Med. Rehabil..

[55] Nordmark E., Andersson G. (2002). Wartenberg pendulum test: Objective quantification of muscle tone in children with spastic diplegia undergoing selective dorsal rhizotomy. Dev. Med. Child Neurol..

[56] Shumway-Cook A., Woollacott M.H. (1985). Dynamics of postural control in the child with Down syndrome. Phys. Ther..

[57] Gillies A.R., Lieber R.L. (2011). Structure and function of the skeletal muscle extracellular matrix. Muscle Nerve.

[58] Gordon M.K., Hahn R.A. (2010). Collagens. Cell Tissue Res..

[59] Korenberg J.R., Chen X.N., Schipper R., Sun Z., Gonsky R., Gerwehr S., Carpenter N., Daumer C., Dignan P., Disteche C. (1994). Down syndrome phenotypes: The consequences of chromosomal imbalance. Proc. Natl. Acad. Sci. USA.

[60] Aimola E., Valle M.S., Casabona A. (2014). Effects of predictability of load magnitude on the response of the Flexor Digitorum Superficialis to a sudden fingers extension. PLoS ONE.

[61] de Azevedo E.R., Maria R.M., Alonso K.C., Cliquet A. (2015). Posture Influence on the Pendulum Test of Spasticity in Patients with Spinal Cord Injury. Artif. Organs.

[62] Willaert J., Desloovere K., Van Campenhout A., Ting L.H., De Groote F. (2020). Movement History Influences Pendulum Test Kinematics in Children With Spastic Cerebral Palsy. Front. Bioeng. Biotechnol..

